# Net Ultrafiltration Rate and Mortality in Critically Ill Patients: A Multicenter Cohort Study With Analysis of Fluid Balance Changes

**DOI:** 10.1097/CCM.0000000000007289

**Published:** 2026-08-12

**Authors:** Nadine C. Monai, Carmen A. Pfortmueller, Joerg C. Schefold, Luca Cioccari, Martin Faltys

**Affiliations:** 1 Department of Intensive Care Medicine, University Hospital, University of Bern, Bern, Switzerland.; 2 Department of Intensive Care Medicine, Kantonsspital Aarau, Aarau, Switzerland.

**Keywords:** continuous renal replacement therapy, critical care, critical illness/mortality, ultrafiltration, water-electrolyte balance

## Abstract

**OBJECTIVES:**

To investigate the association between net ultrafiltration (NUF) rate and fluid balance changes with 28-day mortality in critically ill patients receiving continuous renal replacement therapy (CRRT).

**DESIGN:**

Multicenter retrospective observational study.

**SETTING:**

Data from two European mixed medical-surgical ICUs: University Hospital Bern, Switzerland, and Amsterdam University Medical Center, the Netherlands.

**Patients:**

Adult critically ill patients receiving CRRT for at least 24 hours.

**INTERVENTIONS:**

None.

**MEASUREMENTS AND MAIN RESULTS:**

We studied 973 critically ill adults undergoing a total of 6914 days of CRRT. NUF rates and fluid balance changes were calculated from hourly data and categorized into four predefined groups. NUF rates were classified as no NUF, low (< 1.01 mL/kg/h), moderate (1.01–1.75 mL/kg/h), or high (> 1.75 mL/kg/h). Fluid balance change was stratified as negative (< −1.5 L/day), moderate negative (−1.5 to −0.5 L/day), neutral (−0.5 to 0.5 L/day), or positive (> 0.5 L/day). Using multivariable regression, random forest modeling, and mediation analysis, we observed no independent association between NUF rate and 28-day mortality after accounting for fluid balance. Mediation analysis suggested that the apparent survival advantage observed among patients with higher NUF rates was largely mediated through achievement of a more negative fluid balance, rather than a direct effect of NUF rate itself. In contrast, more positive fluid balance changes were linked to adverse clinical outcomes including 28-day mortality.

**CONCLUSIONS:**

In this two-center cohort of CRRT patients, NUF rate was not independently associated with mortality. Instead, the key determinant of outcome was fluid balance change. These findings suggest clinicians should not make NUF decisions in isolation, but rather should be using NUF as a means to achieve patient-specific fluid balance targets.

**KEY POINTS**

**Question:** Is net ultrafiltration (NUF) rate independently associated with 28-day mortality in critically ill patients receiving continuous renal replacement therapy (CRRT), or is any observed association mediated by fluid balance?

**Findings:** In this European multicenter cohort of 973 CRRT patients, NUF rate was not independently associated with 28-day mortality. In contrast, more positive fluid balance was consistently associated with higher mortality. Mediation analysis suggested that the apparent survival benefit of higher NUF rates was largely explained by achieving a more negative fluid balance.

**Meaning:** These observational data suggest that achieved fluid balance may be more closely associated with outcomes than ultrafiltration intensity alone, highlighting the need for prospective studies to guide fluid removal strategies in CRRT.

Critically ill patients in the ICU frequently develop life-threatening organ dysfunction requiring advanced monitoring and organ support (1). Acute kidney injury (AKI) is particularly common, affecting up to 50% of ICU patients, with approximately one-third requiring renal replacement therapy (2).

Up to two-thirds of ICU patients with AKI develop fluid accumulation syndrome (FAS), typically defined as a cumulative positive fluid balance exceeding 5–10% of body weight (3–5). FAS is independently associated with worse outcomes, including prolonged mechanical ventilation, longer ICU stays, and increased mortality (6–9). Therefore, effective fluid management, including both fluid resuscitation and removal, has become central to ICU care.

When fluid overload becomes resistant to diuretics, continuous renal replacement therapy (CRRT) is often required (10, 11). The key parameter guiding fluid removal during CRRT is the net ultrafiltration (NUF) rate, defined as the effective amount of fluid removed per kilogram of body weight per hour (12). Higher NUF rates are likely to result in more negative fluid balances, potentially mitigating fluid accumulation with associated harmful effects. However, excessive ultrafiltration may lead to intravascular fluid depletion, hemodynamic instability, and impaired organ perfusion (13).

The optimal NUF rate in critically ill patients receiving CRRT remains uncertain. Clinical practice varies widely due to the absence of robust, evidence-based guidelines (14, 15). Murugan et al (3) observed increased mortality with low NUF rates (1.04 mL/kg/h), whereas a follow-up analysis (16) reported that high NUF rates (> 1.75 mL/kg/h) were also linked to increased mortality, suggesting a U-shaped association between NUF intensity and outcomes. Other observational studies (17, 18)—primarily from Australia and New Zealand and often based on overlapping datasets—identified moderate NUF rates (1.01–1.75 mL/kg/h) as ideal. More recent studies from the United States (19) and China (20) reported divergent findings, with some identifying upper thresholds of benefit extending to 3.1 mL/kg/h.

A recent systematic review and meta-analysis observed no consistent association between NUF intensity and mortality across all critically ill patients. However, substantial heterogeneity across studies limited the strength of its conclusions (21). Notably, most studies lacked data on fluid balance, which may mediate or confound observed associations.

Given these limitations, we aimed to investigate the association between NUF rate and mortality in critically ill patients receiving CRRT, with a particular focus on whether this association is mediated by achieved fluid balance rather than ultrafiltration intensity itself.

## MATERIALS AND METHODS

We conducted a multicenter retrospective observational cohort study using two European ICU datasets: Bern University Hospital Bern, Switzerland (2013–2019) (22), referred to as high time resolution ICU data set (HiRID), and Amsterdam University Medical Centers Database (AmsterdamUMCDb) (2003–2016) (23). Ethics approval was obtained from the relevant human research committee (Kantonale Ethikkommission, Canton of Bern approval number 2020-01463, Title: FLuid management in ICU patients receiving Renal Replacement Therapy FLIRRT, approval date: February 25, 2020) with a waiver for written informed consent. The study procedures were conducted in accordance with the ethical standards of the responsible institutional committees and with the Declaration of Helsinki, as most recently amended.

We extracted demographics, severity scores, CRRT parameters, fluid balance data, vital signs, vasopressor doses, lactate levels, and clinical outcomes. For the HiRID population, complete diagnosis lists were independently reviewed by two clinicians to identify patients with sepsis, AKI, and end-stage kidney disease (ESKD, including kidney transplantation). Discrepancies were resolved by consensus through discussion. No imputation was applied as patients missing key parameters (weight and outcome data) were excluded.

### Population

We included adult patients (≥ 18 y) admitted to the ICU and treated with any modality of CRRT for any indication. Only the first session of CRRT was considered. A CRRT session ended after a treatment pause exceeding 24 hours. Patients were excluded if they died within 24 hours of ICU admission (to reduce competing risk), received less than 24 hours of CRRT, had unknown 28-day outcome, or had missing body weight data.

### Outcomes and Definitions

The main objective was to evaluate the association between NUF rate and 28-day mortality. The primary exposure was hourly NUF rate (mL/kg/h), calculated from CRRT machine data normalized by admission body weight. According to previous studies (17, 18), NUF rates were categorized as “low” 0.01–1.0 mL/kg/h, “moderate” 1.01–1.75 mL/kg/h, and “high” greater than 1.75 mL/kg/h, with an additional category of “zero” for patients who never received any NUF throughout their CRRT session.

The second exposure variable was hourly fluid balance change, calculated independently for each hour by summing all fluid inputs and outputs contained in the high-resolution data, including but not limited to the CRRT circuit. Implausible values exceeding ± 10,000 mL/h were removed. Fluid balance changes were stratified into “negative” less than –62.5 mL/h (< –1.5 L/day), “moderate negative” –62.5 to –20.84 mL/h (–1.5 to –0.51 L/day), “neutral” –20.83 to 20.83 mL/h (–0.5 to 0.5 L/day), and “positive” greater than 20.83 mL/h (> 0.5 L/day). We removed outliers with a *z*-score larger than 1.96 across all available fluid balance changes and manually validated the approach using distribution plots.

For each patient, we calculated both the mean value over the full treatment duration and the upper quartile (Q3) value. The Q3 quartile represents the highest ultrafiltration rates and fluid balance changes sustained for at least 25% of the CRRT duration, capturing exposure to higher treatment intensities. Sensitivity analyses also consider mean values during the first 48 hours of CRRT, mean during periods with positive NUF rate or negative fluid balance change, and the early increase in NUF rate, defined as the total volume removed during the first 12 hours of treatment.

### Statistical Analysis

Categorical and continuous variables were compared using chi-square, Wilcoxon rank-sum, or *t*-tests. Spline plots and heatmaps visualized associations between exposures and mortality. Multivariable logistic regression included NUF group and fluid balance group, adjusted for age, sex, body mass index, emergency admission, Acute Physiology and Chronic Health Evaluation (APACHE) II, Sequential Organ Failure Assessment (SOFA) at CRRT start, CRRT duration, and mechanical ventilation. A sensitivity analysis additionally adjusted for noradrenaline dose increase greater than 0.1 µg/kg/min.

To evaluate whether the NUF–mortality association was independent of fluid balance, we fitted logistic regression models with NUF rate as the sole exposure variable, both unadjusted and adjusted for covariates, and compared these with models additionally including fluid balance change. Potential collinearity between NUF rate and fluid balance was assessed using generalized variance inflation factors (GVIF) and Spearman correlation.

Causal mediation analysis estimated direct and indirect (via fluid balance) effects of NUF on mortality, with 95% CIs from 1000 bootstrap simulations. Random forest models with partial dependence and three-dimensional surface plots explored nonlinear relationships.

To address potential survivor bias, we performed landmark analyses at 48 and 72 h after CRRT initiation. Only patients alive at each timepoint were included, and exposures were calculated as mean values during the landmark window only. Cox proportional hazards models assessed associations between these fixed early exposures and subsequent 28-day mortality, with the proportional hazards assumption evaluated using Schoenfeld residuals.

Sensitivity analyses included subgroups by hospital, sex, pre-CRRT fluid overload, vasopressor and lactate trends, anuria, sepsis, and AKI. Alternative exposure definitions included upper quartile (Q3), first 48 hours, positive-NUF-only periods, and early NUF increase. All subgroup and sensitivity analyses were exploratory and not adjusted for multiple comparisons; results should be interpreted as hypothesis-generating. All preprocessing used Python 3.8 (Python Software Foundation, Beaverton, OR) (24). R (R Foundation for Statistical Computing, Vienna, Austria) (25) was used for statistical analysis. The code including statistical analysis and all used libraries can be found on https://github.com/mafacz/2021_FLIRRT.

## RESULTS

Of 1263 critically ill patients treated with CRRT at the two European university hospitals, we included 973 patients (6914 CRRT treatment days). The most common exclusion was CRRT duration less than 24 h (*n* = 209; **Fig. 1**). Of the included patients, 495 patients (50.9%) were from the HiRID database and 478 patients (49.1%) from the AmsterdamUMCDb. The median age was 64 years (interquartile range [IQR], 54.0–74.0 yr), and one-third of patients were female (**Table 1**, stratified by mean NUF group in **eTable 1**, https://links.lww.com/CCM/I30). Compared with AmsterdamUMCdb, HiRID patients were more often emergency admissions (84% vs. 41%) and had higher severity of illness at ICU admission and at CRRT initiation. At the start of CRRT, 84.3% had a positive cumulative fluid balance and 41.8% of patients had fluid overload (≥ 7% of body weight). Baseline characteristics were largely consistent across NUF groups (eTable 1, https://links.lww.com/CCM/I30), including severity of illness as assessed by SOFA and APACHE scores. The high NUF group had the highest rate of emergency admissions, as well as severity of illness scores at admission (APACHE II) and start of CRRT (SOFA), but the lowest vasopressor requirements and lactate levels. Most patients of the HiRID cohort suffered from AKI, while only 6.1% needed CRRT due to ESKD. More than 50% of HiRID patients had a sepsis diagnosis, while 35% of all patients were surgical admissions (Table 1).

**TABLE 1.** Baseline Characteristics for the Overall Cohort and by Database

		Database	
Characteristic	Overall *n* = 973^a^	Amsterdam University Medical Center *n* = 478^a^	High Time Resolution ICU Data Set *n* = 495^a^
Female sex	328 (33.7%)	155 (32.4%)	173 (34.9%)
Age (yr)	64.0 (54.0–74.0)	64.0 (54.0–74.0)	64.0 (55.0–72.0)
Height (cm)	174 (165–180)	174 (164–184)	170 (165–180)
Weight (kg)	80 (70–94)	84 (74–94)	80 (70–90)
Body mass index (kg/m^2^)	26.8 (23.9–30.7)	27.5 (23.8–30.7)	26.5 (23.9–30.5)
Emergency admission	611 (62.8%)	196 (41.0%)	415 (83.8%)
Reason for ICU admission			
Cardiovascular or vascular	242 (24.9%)	82 (17.2%)	160 (32.3%)
Respiratory	86 (8.8%)	10 (2.1%)	76 (15.4%)
Gastrointestinal	65 (6.7%)	15 (3.1%)	50 (10.1%)
Other medical	211 (21.7%)	150 (31.4%)	61 (12.3%)
Cardiovascular or vascular surgery	219 (22.5%)	122 (25.5%)	97 (19.6%)
Other surgical	59 (6.1%)	39 (8.2%)	20 (4.0%)
Gastrointestinal surgery	60 (6.2%)	29 (6.1%)	31 (6.3%)
Unknown	31 (3.2%)	31 (6.5%)	0 (0.0%)
APACHE II Score at ICU admission	25 (20–31)	22 (19–25)	30 (24–34)
SOFA Score at start of CRRT	11 (9–14)	10 (8–12)	13 (10–15)
Invasive ventilation at start of CRRT	791 (81.3%)	370 (77.4%)	421 (85.1%)
Noradrenaline at start of CRRT (µg/kg/min)	0.1 (0.0–0.3)	0.2 (0.0–0.6)	0.0 (0.0–0.1)
Lactate at start of CRRT (mmol/L)	2.6 (1.5–5.3)	2.2 (1.4–4.5)	2.9 (1.6–6.7)
Acute kidney injury	465 (93.9%)	0 (NA%)	465 (93.9%)
End-stage kidney disease	30 (6.1%)	0 (NA%)	30 (6.1%)
Postsurgical acute kidney injury	141 (28.5%)	0 (NA%)	141 (28.5%)
Sepsis	259 (52.3%)	0 (NA%)	259 (52.3%)
Fluid balance before CRRT (L)	6.9 (8.6)	8.7 (8.8)	5.0 (8.0)
Positive fluid balance before CRRT	799 (84.3%)	425 (88.9%)	374 (79.6%)
Fluid overload ≥ 5% (kg body weight)	489 (50.3%)	272 (56.9%)	217 (43.8%)
Fluid overload ≥ 7% (kg body weight)	407 (41.8%)	235 (49.2%)	172 (34.7%)
Fluid overload ≥ 10% (kg body weight)	273 (28.1%)	174 (36.4%)	99 (20.0%)
ICU length of stay (d)	10.5 (5.8–20.2)	12.0 (6.2–27.0)	8.8 (5.5–16.1)
ICU death	368 (37.8%)	184 (38.5%)	184 (37.2%)
28-D death	377 (38.7%)	163 (34.1%)	214 (43.2%)

APACHE = Acute Physiology and Chronic Health Evaluation, CRRT = continuous renal replacement therapy, SOFA = Sequential Organ Failure Assessment.

a *n* (%); median (Q1–Q3); mean (sd).

**Figure 1.**
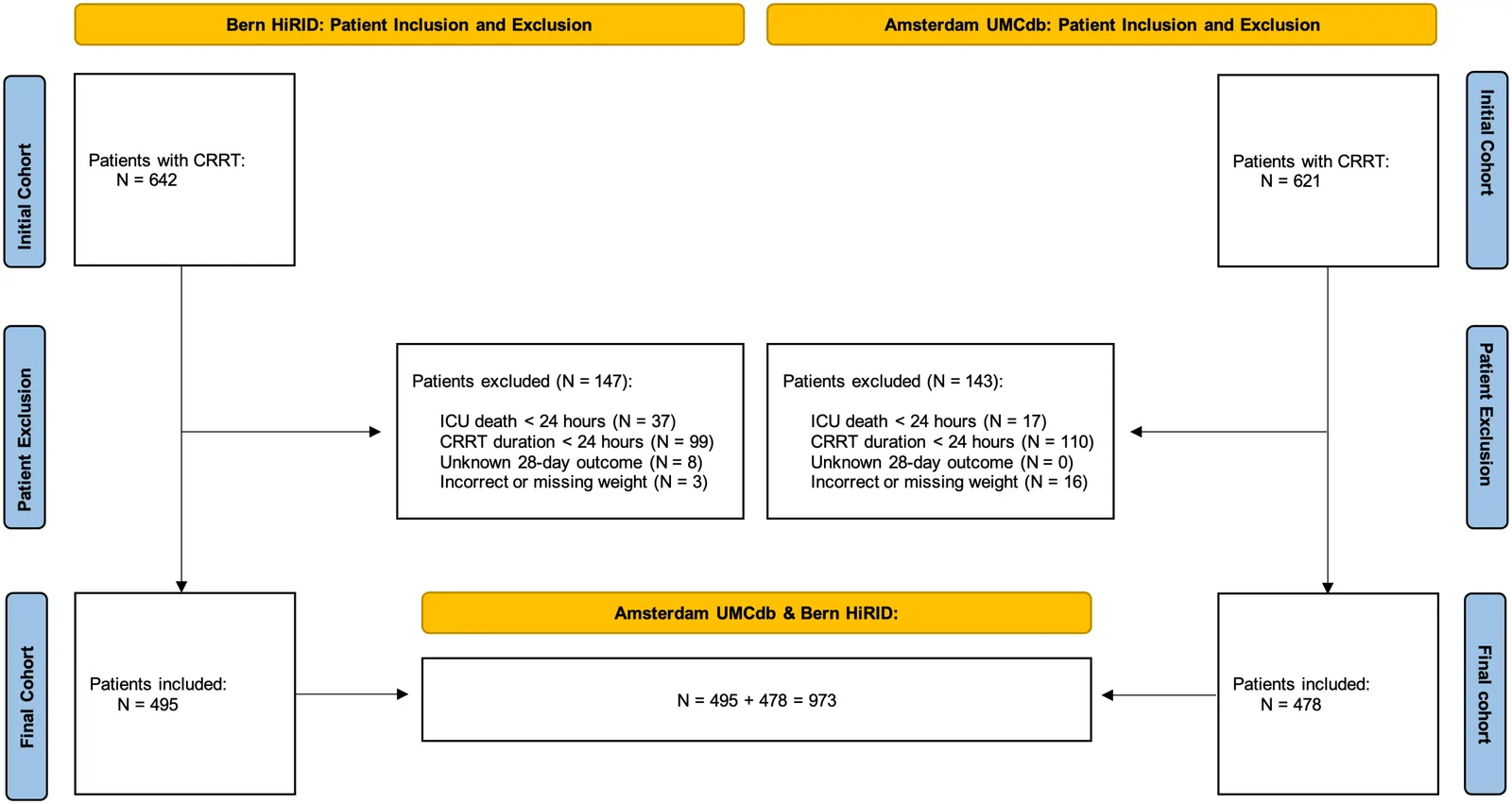
Flowchart of patient inclusion and exclusion. CRRT = continuous renal replacement therapy, HiRID = high time resolution ICU data set, UMCdb = University Medical Centers Database.

There were substantial differences in CRRT practice between cohorts (**Table 2**; **eFig. 1,** https://links.lww.com/CCM/I30). Patients in Bern received continuous venovenous hemodiafiltration (CVVHDF) with higher NUF rates achieving more negative fluid balances, while Amsterdam patients predominantly received continuous venovenous hemofiltration (CVVH) with lower NUF rates. Notably, fewer than 1% of patients in Amsterdam received high NUF rates, compared with 14% in HiRID. Despite shorter CRRT durations (3.8 vs. 5.2 d), 68% of HiRID patients achieved a negative cumulative fluid balance, compared with 28% in AmsterdamUMCdb.

**TABLE 2.** Continuous Renal Replacement Therapy Modality, Ultrafiltration Intensity, and Fluid Balance in the Overall Cohort and by Database

		Database	
Characteristic	Overall *n* = 973^a^	Amsterdam University Medical Center *n* = 478^a^	High Time Resolution ICU Data Set *n* = 495^a^
Type of continuous renal replacement therapy			
CVVH	375 (39%)	375 (79%)	0 (0%)
HVCVVH	99 (10%)	99 (21%)	0 (0%)
CVVHDF	495 (51%)	0 (0%)	495 (100%)
Duration of CRRT (d)	4.4 (2.6–7.9)	5.2 (2.8–11.3)	3.8 (2.3–6.5)
Time until start of CRRT from ICU admission (d)	2 (1–6)	4 (2–14)	1 (0–2)
Time from start of CRRT until ultrafiltration (hr)	14 (1–33)	14 (1–30)	14 (1–35)
Mean ultrafiltration rate (mL/kg/hr)	0.73 (0.64)	0.51 (0.38)	0.94 (0.76)
Group of mean ultrafiltration rate (mL/kg/hr)			
Zero (0)	117 (12%)	60 (13%)	57 (12%)
Low (0.01–1.0)	601 (62%)	372 (78%)	229 (46%)
Moderate (1.01–1.75)	181 (19%)	42 (8.8%)	139 (28%)
High (> 1.75)	74 (7.6%)	4 (0.8%)	70 (14%)
Percentage of time with ultrafiltration = 0	44 (31)	41 (32)	47 (30)
Patients without ultrafiltration > 0 during entire CRRT	117 (12%)	60 (13%)	57 (12%)
Mean hourly change of fluid balance (mL/hr)	–48 (125)	–9 (62)	–86 (155)
Group of mean fluid balance change (mL/hr)			
Positive (> 20.83)	210 (22%)	121 (25%)	89 (18%)
Neutral (–20.83 to 20.83)	180 (18%)	126 (26%)	54 (11%)
Moderate negative (–62.5 to –20.84)	227 (23%)	162 (34%)	65 (13%)
Negative (< –62.5)	356 (37%)	69 (14%)	287 (58%)
Negative cumulative fluid balance at the end of CRRT	452 (48%)	132 (28%)	320 (68%)
Negative total fluid change during CRRT	602 (62%)	242 (51%)	360 (73%)
Total fluid change during CRRT per d (L/d)	–0.6 (–3.0 to 1.0)	0.0 (–0.8 to 1.4)	–2.8 (–5.6 to 0.3)
Total fluid removed by CRRT per d (L/d)	–1.20 (–2.06 to –0.50)	–1.00 (–1.46 to –0.38)	–1.75 (–2.83 to –0.62)
Relative fluid balance change (% of fluid balance before CRRT) in patients with positive fluid balance	–36 (–260 to 72)	4 (–66 to 104)	–188 (–587 to 12)
Percentage of time with negative hourly fluid balance change during CRRT (%)	56 (21)	53 (24)	59 (17)
Percentage of time with negative hourly FB change when ultrafiltration > 0 (%)	74 (16)	73 (18)	75 (14)

CRRT = continuous renal replacement therapy; CVVH = continuous venovenous hemofiltration; CVVHDF = continuous venovenous hemodiafiltration; FB = fluid balance; HVCVVH = high-volume continuous venovenous hemofiltration.

a *n* (%); median (Q1–Q3); mean (sd)

Median ICU stay was 10.5 days (IQR, 5.8–20.2 d), longer in AmsterdamUMCdb. Finally, ICU mortality was similar across cohorts, but 28-day mortality was higher in HiRID (43% vs. 34%; Table 1).

### Association of Net Ultrafiltration Rate and Fluid Balance Change with 28-Day Mortality

Smoothed spline plots and stratified heatmaps showed highest 28-day mortality at low NUF rates. The “Zero” NUF group had the highest crude 28-day mortality at 79%, compared with an overall mortality of 39% (**Fig. 2*C***). Mortality remained consistently lower across increasing NUF rates with no evidence of a U- or J-shaped curve (**Fig. 2*A***). Similarly, high NUF exposure (Q3) was not associated with increased mortality. In contrast, more positive fluid balance changes were clearly associated with higher mortality, while more negative balances were protective (**Fig. 2*B***). These findings remained consistent in sensitivity analyses (**eFig. 2**, https://links.lww.com/CCM/I30) limited to the first 48 hours of treatment or to timepoints with actual ultrafiltration.

**Figure 2.**
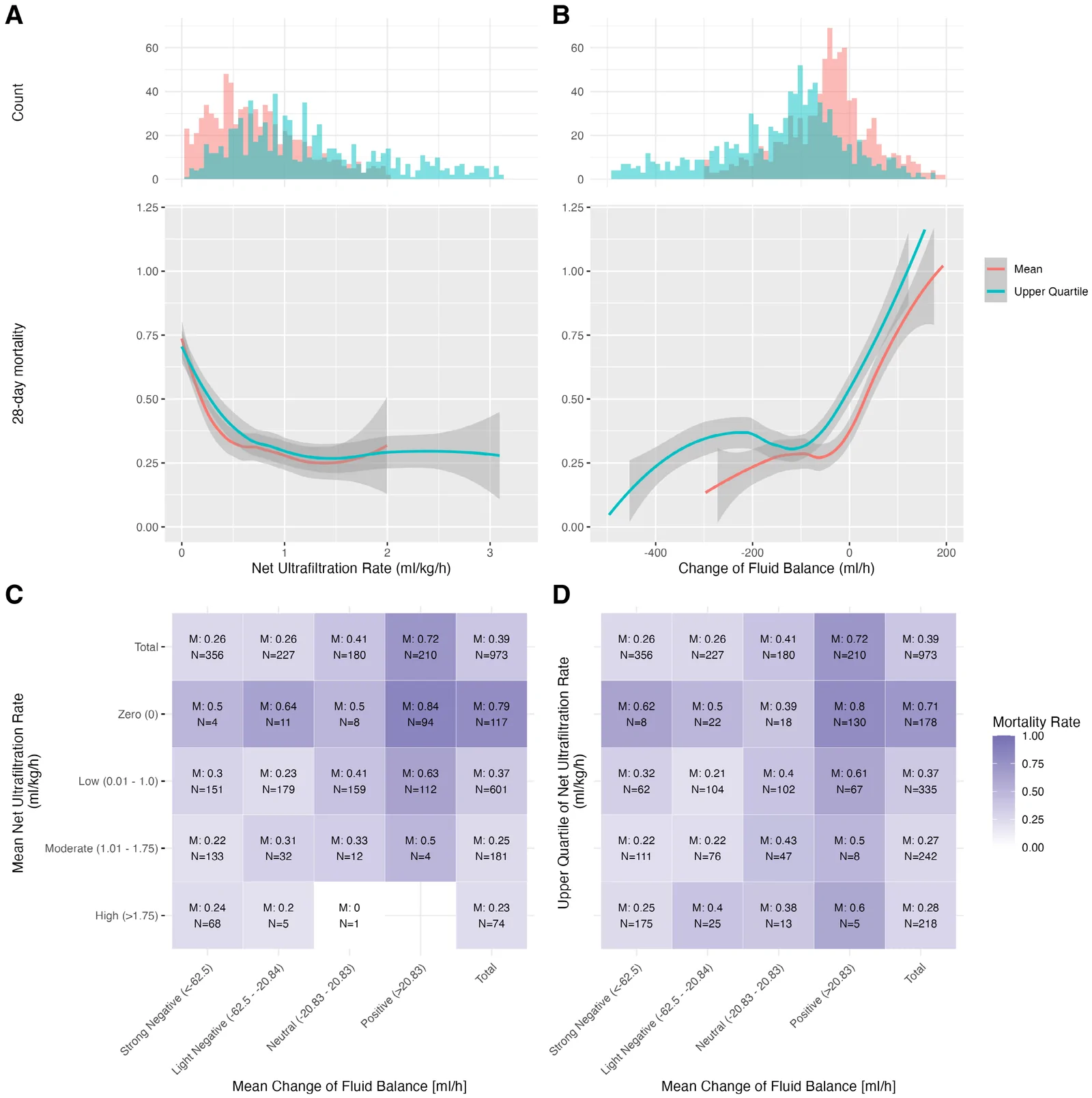
Relationship between net ultrafiltration (NUF) rate, fluid balance change, and 28-day mortality. **A**, Smoothed spline plot of NUF rate (mL/kg/h) and 28-d mortality. **B**, Smoothed spline plot of fluid balance change (mL/h) and 28-d mortality. **C**, Heatmap of crude 28-d mortality stratified by mean NUF rate and mean fluid balance change. **D**, Heatmap of crude 28-d mortality stratified by upper quartile of NUF rate and mean fluid balance change. *Shaded areas* represent 95% CIs. Histograms display the distribution of NUF rates and fluid balance changes. *Red lines* indicate mean rates per patient; *blue lines* indicate upper quartile rates per patient.

In logistic regression models with NUF rate as sole exposure, lower NUF rates were significantly associated with higher mortality (low vs. moderate: odds ratio [OR], 2.24; *p* < 0.001; zero vs. moderate: OR, 10.68; *p* < 0.001; **eFig. 3 *A*–*B***, https://links.lww.com/CCM/I30). After adding fluid balance, these associations were substantially attenuated (low: OR, 1.26; *p* = 0.280; zero: OR, 3.81; *p* < 0.001) (**eFig. 3*C***, https://links.lww.com/CCM/I30). GVIF values for NUF rate and fluid balance were 1.81 and 1.91, confirming this attenuation was not driven by collinearity. The Spearman correlation between NUF rate and fluid balance was −0.72 (*p* < 0.001), consistent with the hypothesized mediation pathway.

In the fully adjusted model including both exposures (**Fig. 3**), fluid balance change was a strong independent predictor. A strongly negative fluid balance was associated with lower mortality (OR, 0.41; 95% CI, 0.26–0.66; *p* < 0.001), whereas a positive fluid balance was associated with increased mortality (OR, 2.05; 95% CI, 1.26–3.36; *p* = 0.004), both relative to the neutral fluid balance group. Twenty-three patients had missing APACHE score at admission and were not included in the model.

**Figure 3.**
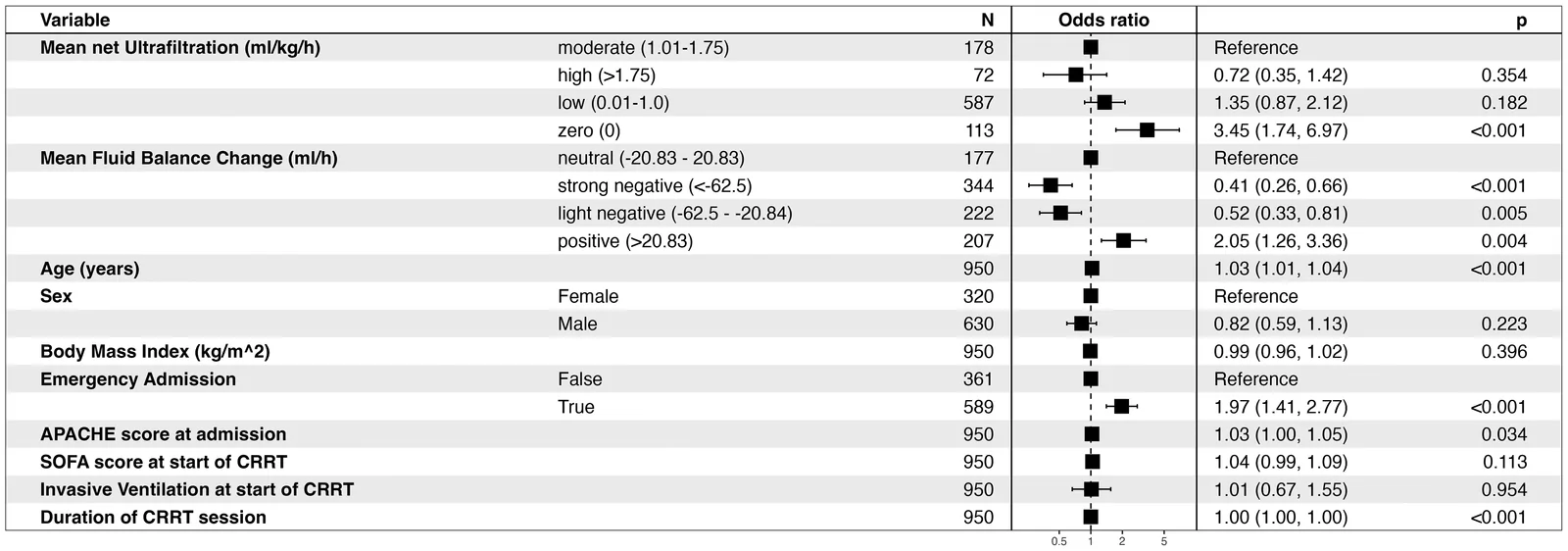
Multivariate logistic regression model of mean net ultrafiltration rate (NUF) and fluid balance change. Adjusted logistic regression analysis assessing the association between categorized mean NUF rate and fluid balance change with 28-d mortality. Estimates are adjusted for demographic and clinical confounders. APACHE = Acute Physiology and Chronic Health Evaluation, CRRT = continuous renal replacement therapy, SOFA = Sequential Organ Failure Assessment.

These findings were consistent in sensitivity analyses using alternative NUF summary metrics and time windows (**eFig. 4 *A*–*C***, https://links.lww.com/CCM/I30), including noradrenaline increase (**eFig. 5*A***, https://links.lww.com/CCM/I30), and analyses of early NUF increase (volume removed in the first 12 hr), which showed stable mortality across different treatment intensities (**eFig. 5*B***, https://links.lww.com/CCM/I30).

In landmark analyses restricted to patients alive at 48 hours (*n* = 705) and 72 hours (*n* = 664) after CRRT initiation, NUF rate was not associated with subsequent mortality at either timepoint, regardless of whether fluid balance was included in the model. At the 72-hour landmark, positive fluid balance was independently associated with increased mortality (hazard ratio [HR], 1.57; 95% CI, 1.04–2.37; *p* = 0.032), while NUF remained nonsignificant (**eTable 2,** https://links.lww.com/CCM/I30). The proportional hazards assumption was satisfied at both timepoints.

### Mediation of Ultrafiltration Effects via Fluid Balance

Prerequisites for mediation were satisfied (**eFig. 6**, https://links.lww.com/CCM/I30): Higher NUF rates were associated with more negative fluid balance changes, and more positive fluid balance changes were associated with higher mortality.

Causal mediation analyses demonstrated that the apparent protective effect of higher NUF was largely mediated through fluid balance change. The average causal mediation effect was significant in all comparisons (except high vs. moderate), with OR ranging from 0.77 to 0.88, and fluid balance mediated up to 89% of the total effect.

Average direct effects (ADE) were only significant when comparing NUF levels to the “zero” NUF group (e.g., moderate vs. zero: ADE OR, 0.71; 95% CI [0.61–0.82]; *p* < 0.001). Total effects (TE) consistently showed ORs less than 1, supporting an association of higher NUF with reduced mortality. No significant effects were found between “high” and “moderate” NUF levels.

Key results are presented in **Figure 4**, complete analyses in **eTable 3** (https://links.lww.com/CCM/I30).

**Figure 4.**
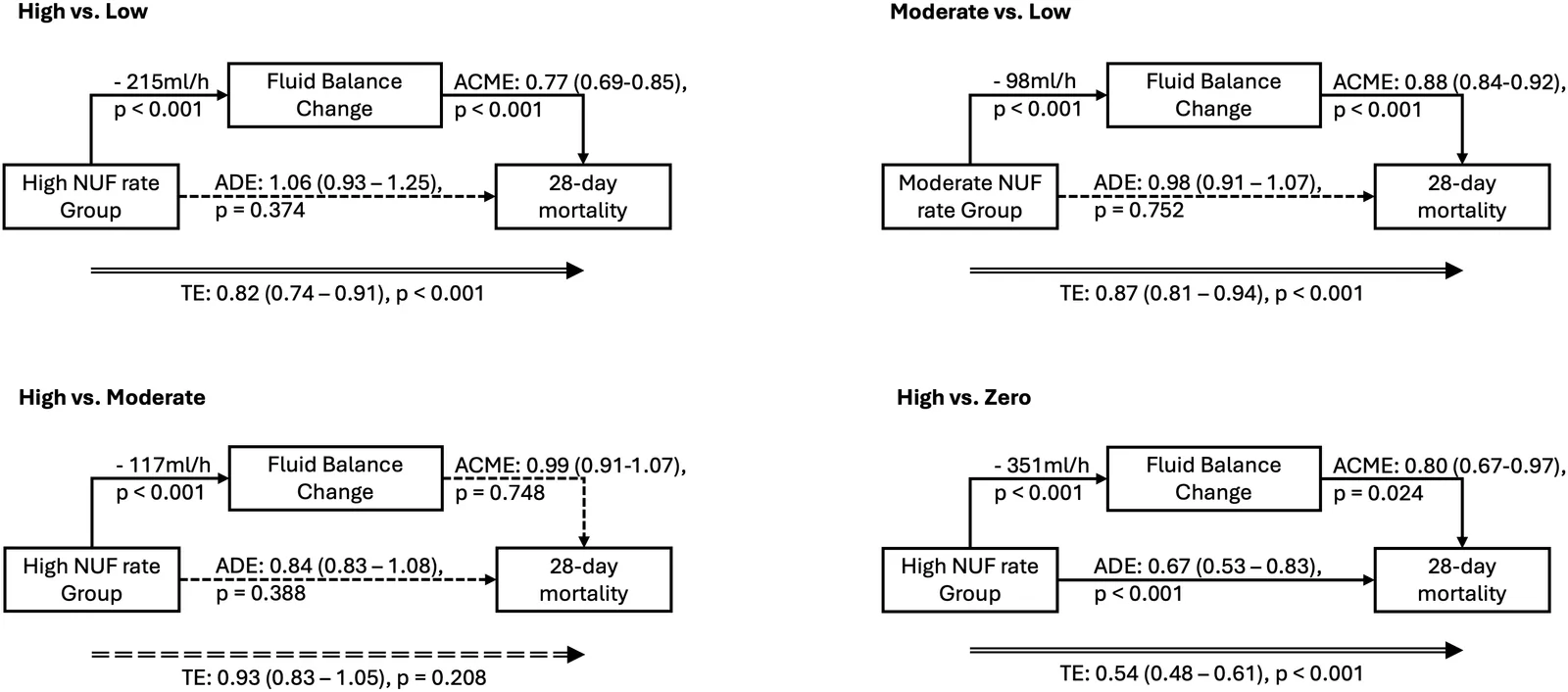
Causal mediation analysis of net ultrafiltration rate (NUF) and fluid balance change. Visual summary of the mediation analysis estimating total, direct, and indirect (mediated) effects of NUF rate on 28-d mortality through fluid balance change. *Dashed lines* represent nonsignificant results. ACME = average causal mediation effect, ADE = average direct effects, TE = total effects.

### Robustness Analyses: Dependence Plots and Subgroup Analyses

These findings were further supported by partial dependence plots, showing no increase in 28-day mortality with higher NUF rates (**eFig. 7*A***, https://links.lww.com/CCM/I30) and a clear increase in mortality with more positive fluid balance **(eFigs. 7*B*** and **8**, https://links.lww.com/CCM/I30).

Subgroup analyses showed no differences by data source (**eFig. 9**, https://links.lww.com/CCM/I30), sex, pre-CRRT fluid overload (≥ 10%), noradrenaline increase greater than or equal to 0.1 mg/kg/min, or lactate increase greater than or equal to 2 mmol/L during CRRT (eFig. 9, **eTable 4**, https://links.lww.com/CCM/I30). However, a U-shaped association emerged for patients with lactate increase greater than or equal to 4 mmol/L (**eFig. 10*E***, https://links.lww.com/CCM/I30) and those with new onset of anuria during CRRT (**eFig. 1*0F***, https://links.lww.com/CCM/I30), although data were sparse in these subgroups. In addition, subgroup analyses restricted to HiRID patients with AKI and to those with a diagnosis of sepsis were consistent with our primary findings (**eFig. 11, *A*** and ***B***, https://links.lww.com/CCM/I30). The subgroup of ESKD was not separately analyzed due to insufficient number of patients (*n* = 30).

## DISCUSSION

### Main Findings

In this retrospective study of 973 critically ill patients receiving CRRT in two European university hospitals, NUF rate was not independently associated with 28-day mortality. In contrast, a positive fluid balance change was consistently associated with higher mortality. The benefit of higher NUF rates was largely mediated by the achievement of more negative fluid balance, not ultrafiltration intensity itself.

### Findings in Context

Our findings suggest that high-rate NUF is not inherently harmful. Previous studies reported increased mortality at higher NUF rates (> 1.75 mL/kg/h), often indicating a U-shaped risk curve (3, 20). In contrast, our findings align with the recent systematic review by Jin et al (21), which found no consistent relationship—although study heterogeneity was significant. However, a potential signal for increased mortality at very high NUF rates cannot be excluded in our cohort due to the limited number of patients exposed to such intensities. Importantly, when NUF rate was modeled without fluid balance, it was significantly associated with mortality, confirming that NUF does have a total effect on outcomes. This effect was substantially attenuated after inclusion of fluid balance change, and collinearity diagnostics (GVIF < 2 for both variables) confirmed that this attenuation reflects genuine mediation rather than model instability due to correlated predictors. Landmark analyses further confirmed that this finding was not driven by survivor bias. Among patients alive at 48 and 72 h after CRRT initiation, early NUF exposure did not predict subsequent mortality, whereas a positive fluid balance during the first 72 h remained significantly associated with worse outcomes. Our work is the first large-scale European multicenter analysis to evaluate NUF and mortality in CRRT patients, expanding generalizability beyond North American and Australasian data and across both CVVH and CVVHDF modalities.

Our results reinforce the hypothesis that fluid balance change, rather than ultrafiltration intensity per se, is the key determinant of patient outcome during CRRT. The association between fluid overload and adverse outcomes is well-established in critically ill patients (6), and our findings extend this concept into the context of CRRT. The dose-response relationship between positive hourly fluid balances and higher mortality is in line with earlier findings from White et al (12). Our causal mediation analysis extends beyond earlier 48-hour windows used by Naorungroj et al (18) and Wu et al (20) and demonstrates that the apparent benefit of higher NUF rates was almost entirely mediated by achieving more negative fluid balance, rather than by ultrafiltration intensity itself. The elevated mortality in the “Zero” NUF group is a novel observation. It may reflect persistent fluid overload and clinical inability to tolerate fluid removal, due to hemodynamic instability. Residual confounding related to clinician decision-making (e.g., indication bias through clinician-imposed limitations) cannot be excluded. More positive fluid balance during CRRT may also reflect a gap between prescribed and delivered ultrafiltration (26), although our cohort focuses on delivered rates. Finally, our findings proved robust across diverse sensitivity analyses, including high-exposure (Q3) and early NUF increase.

### Strengths/Limitations

As an observational study, causal relations cannot be established, and residual confounding remains possible, particularly regarding clinical intent and patient selection for lower NUF rates. Patients with low or zero NUF rates likely represent a heterogeneous group, including those without clinical need for fluid removal, those with hemodynamic intolerance, and those with discordance between prescribed and delivered ultrafiltration. Because detailed prescription data were not consistently available, we focused on delivered ultrafiltration and achieved fluid balance, which reflect the net physiologic exposure. Body weight, used to normalize NUF rates, was not uniformly measured but recorded as part of routine clinical care. Inaccuracies in weight estimation may have led to nondifferential misclassification of NUF intensity, which would be expected to bias associations toward the null. Nevertheless, admission body weight is routinely used to guide multiple ICU therapies, including vasoactive drug dosing, nutrition, and CRRT settings, and is therefore subject to substantial clinical attention. Fluid balance documentation, although comprehensive, may vary in accuracy and completeness. Certain subgroups, such as patients with lactate increases greater than or equal to 4 mmol/L or those exposed to very high NUF rates, were underrepresented for conclusive interpretation. Different ICU populations with distinct fluid balance goals could not be fully differentiated due to limited information regarding the reason for ICU admission. In the AmsterdamUMCdb cohort, clinical diagnoses were not available to reliably distinguish between AKI and ESKD patients requiring CRRT, limiting diagnosis-specific subgroup analyses to HiRID patients only. A marginal structural model with time-varying exposures could further strengthen causal inference. Despite these limitations, this study provides the first large-scale European evaluation of NUF and mortality in CRRT patients, using high-resolution data from two academic centers with comprehensive fluid inputs and outputs. Our analysis combines multivariable regression, causal mediation, random forest models, and extensive sensitivity analyses, with consistent results across modalities, hospitals, and clinical subgroups.

### Implications

Our findings support a nuanced interpretation. NUF rate is associated with mortality, but this association is largely mediated through its effect on fluid balance, making NUF rate alone an insufficient indicator of effective fluid management in critically ill patients on CRRT. Although it reflects the mechanical part of fluid removal, the clinical effect depends on the resulting fluid balance change. In fact, identical NUF values can yield markedly different net fluid balances depending on the concurrent volume of fluid administration. This underscores the need to interpret NUF within the broader context of total fluid balance. Our data suggest that the NUF rate primarily benefits patients when it results in a favorable shift in fluid balance. Thus, clinical decisions about fluid removal should be individualized and guided by patient response, volume tolerance, and physiologic reserve, aligning with the growing emphasis on precision fluid management in the ICU. However, these conclusions are based on observational data, and prospective trials are essential to inform definitive guidelines. The ongoing RELIEVE-AKI trial (27), comparing restrictive vs. liberal volume removal strategies, is a step in this direction. Overall effective fluid management in CRRT may depend less on the intensity of ultrafiltration and more on achieving appropriate, individualized fluid balance.

## CONCLUSIONS

In this large European multicenter study of critically ill patients on CRRT, higher NUF rates were not independently associated with increased mortality, while a positive fluid balance change was a consistent predictor of worse outcome. These findings suggest that fluid balance, not ultrafiltration intensity, is the key mediator linking CRRT to patient mortality. NUF rate may therefore be most useful when titrated to individualized fluid balance goals rather than applied in isolation.

## Supplementary Material
