# Supplementary material for: Net Ultrafiltration Rate and Mortality in Critically Ill Patients: A Multicenter Cohort Study With Analysis of Fluid Balance Changes
Source: Crit Care Med. 2026 Aug 12;54(10):2648–59. doi: 10.1097/CCM.0000000000007289 (PMC13632811; doi:10.1097/CCM.0000000000007289)
Supplement: Supplementary file 1 [file ccm-54-2648-s001.pdf]

Net Ultrafiltration Rate and Mortality in Critically Ill Patients: A Multicenter Cohort Study with Analysis of Fluid Balance Changes

Supplementary Material

Table of Contents

eFigure 1. Distribution of Net Ultrafiltration Rate (NUF) and Fluid Balance Change in Both Cohorts

eFigure 2. Sensitivity Analyses Using Alternative Definitions of NUF and Fluid Balance

eFigure3. Stepwise Logistic Regression Models Demonstrating Attenuation of Net Ultrafiltration Rate (NUF) Effect After Inclusion of Fluid Balance Change.

eFigure 4. Logistic Regression Models Using Alternative Summary Measures of NUF and Fluid Balance

eFigure 5. Logistic Regression including Noradrenalin Increase and for Early Increase in NUF Rate and 28-Day Mortality

eFigure 6. Prerequisite Regression Models for Mediation Analysis

eFigure 7. Partial Dependence Plots (PDP) from Random Forest Models

eFigure 8. Combined Effect of NUF Rate and Fluid Balance Change on Predicted 28-Day Mortality

eFigure 9. Analysis by Database

eFigure 10. Analyses by Different Subgroups

eFigure 11. Analyses by Diagnosis Subgroups

eTable 1: Population Characteristics in different Net Ultrafiltration Groups

eTable 2: Landmark Analyses at 48 and 72 Hours After CRRT Initiation

eTable 3: Mediation Analysis

eTable 4. Vasopressor and Lactate levels and trends, and occurrence of anuria

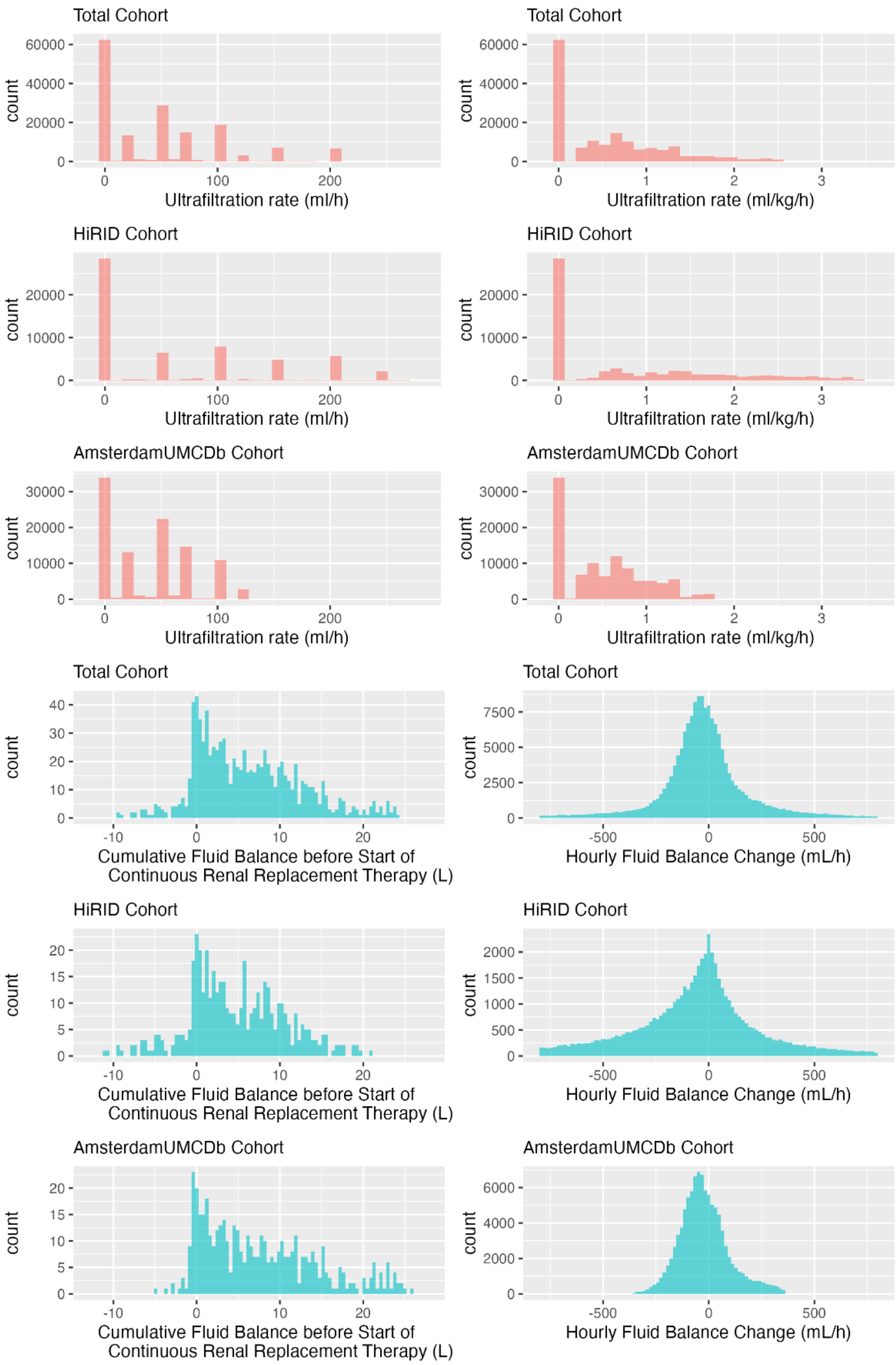

eFigure 1. Distribution of Net Ultrafiltration Rate (NUF) and Fluid Balance Change in Both Cohorts.

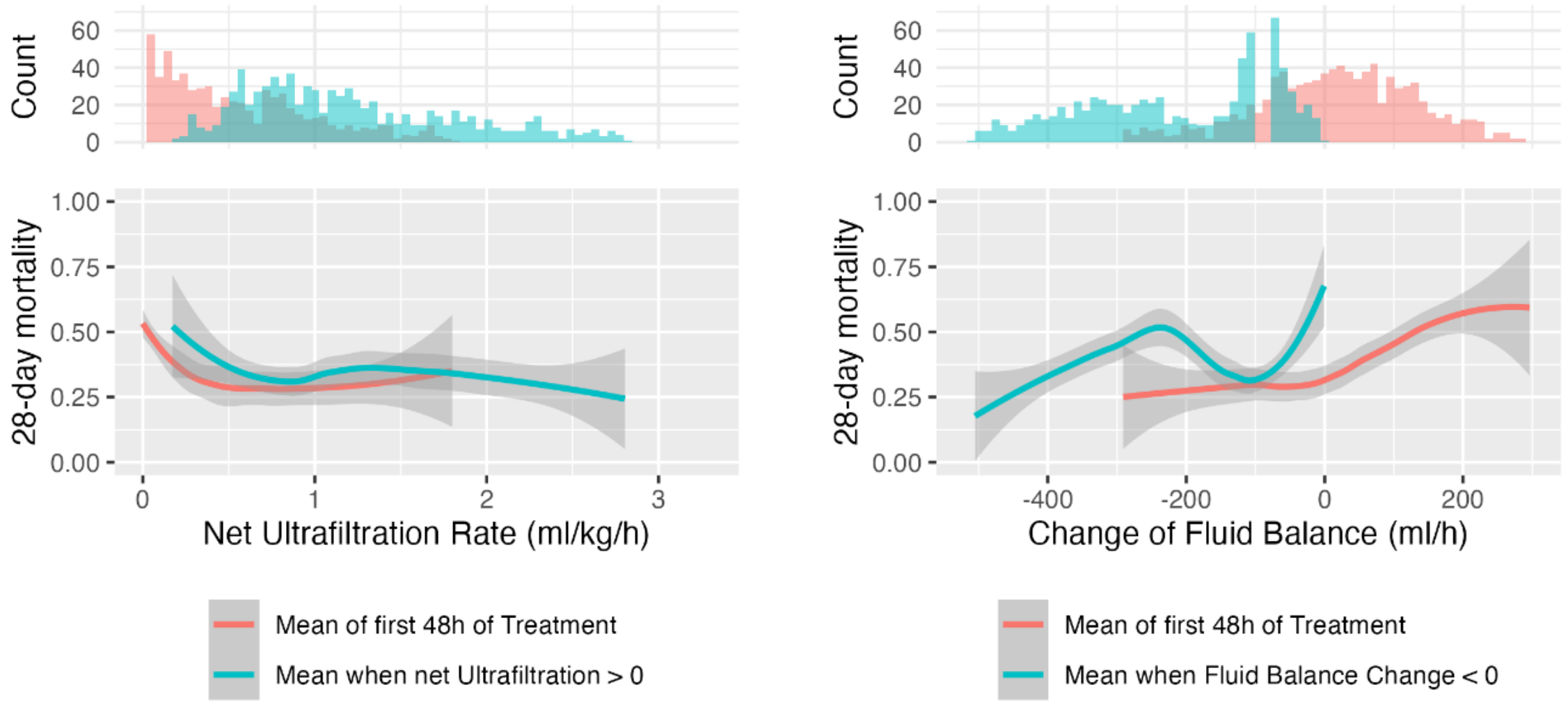

eFigure 2. Sensitivity Analyses Using Alternative Definitions of NUF and Fluid Balance.

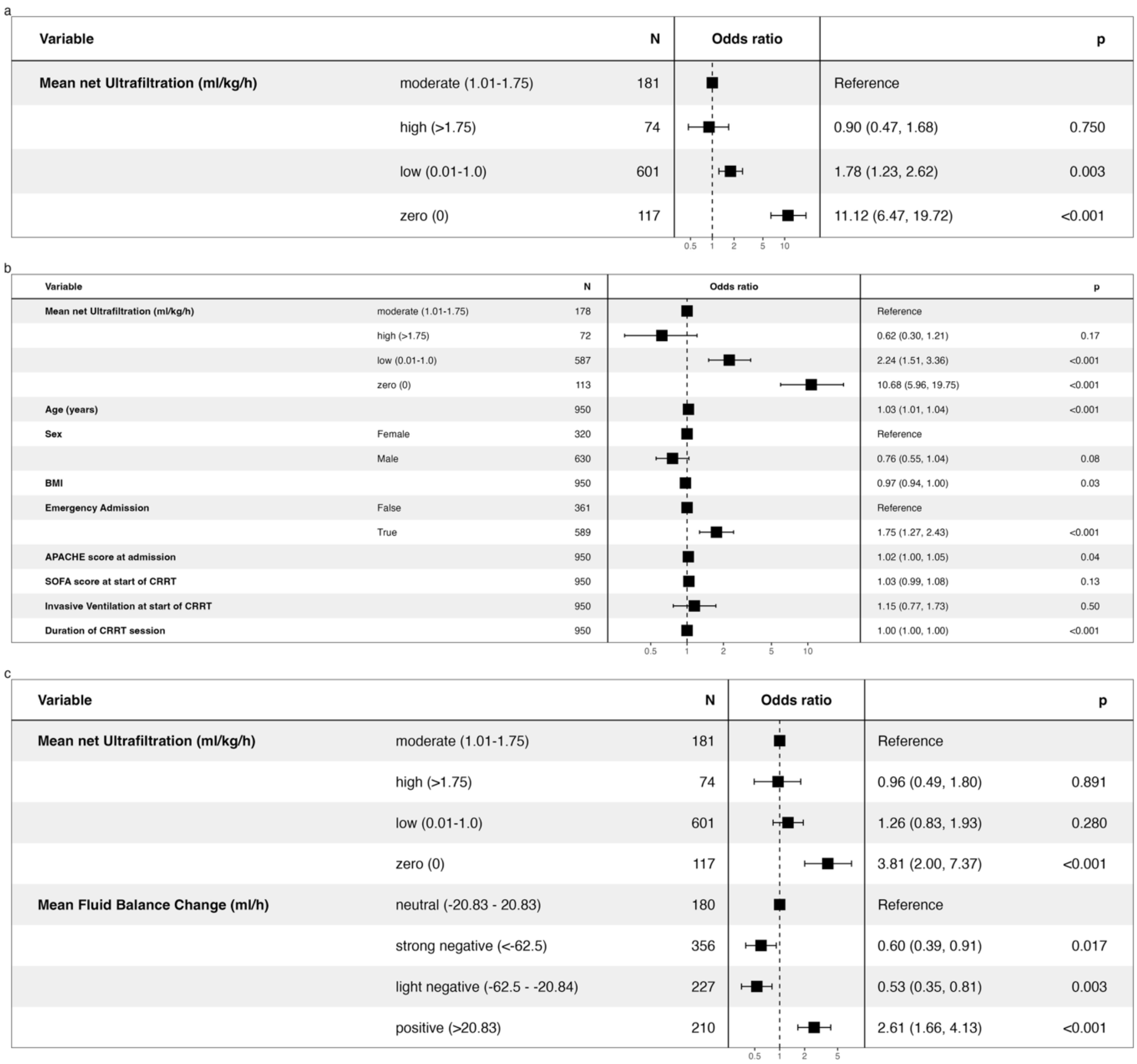

eFigure 3. Stepwise Logistic Regression Models Demonstrating Attenuation of Net Ultrafiltration Rate (NUF) Effect After Inclusion of Fluid Balance Change.

(a) Unadjusted logistic regression model with NUF rate only.
(b) Logistic regression model with NUF rate adjusted for demographic and clinical covariates, without fluid balance change.
(c) Unadjusted logistic regression model including both NUF rate and fluid balance change.

The fully adjusted model including both NUF rate and fluid balance change is presented in Figure 3. Estimates are displayed as odds ratios (OR) with 95% confidence intervals.

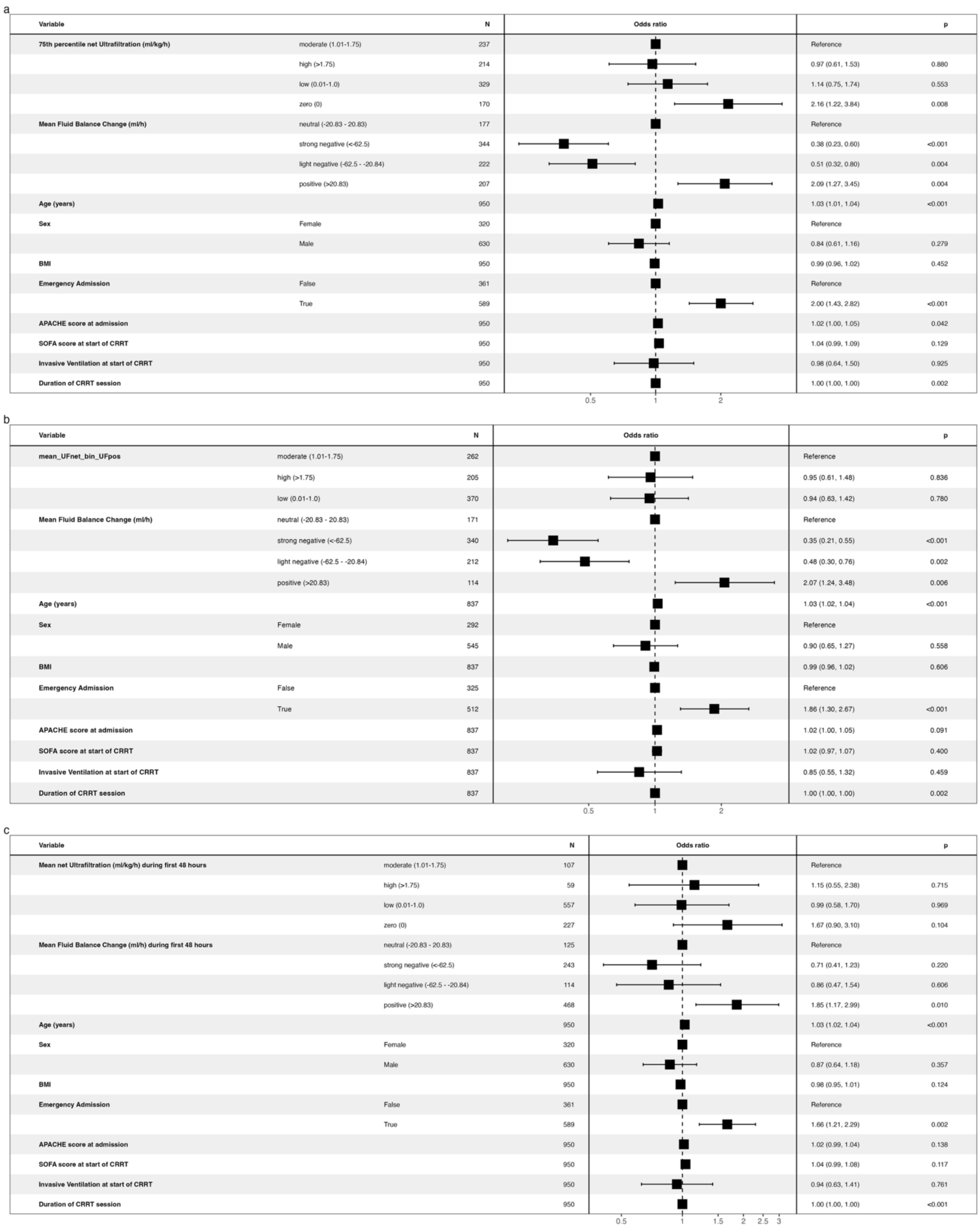

eFigure 4. Logistic Regression Models Using Alternative Summary Measures of NUF and Fluid Balance

(a) Models using upper Quartile of NUF rates per patient
(b) Models using mean NUF during periods with positive NUF rate only.
(c) Models using mean NUF and fluid balance during the first 48 hours.

Estimates are displayed as odds ratios (OR) with 95% confidence intervals.

a

Variable		N	Odds ratio (95% CI)	p
**Mean net Ultrafiltration (ml/kg/h)**	moderate (1.01-1.75)	178	Reference	
	high (>1.75)	72	0.73 (0.36, 1.44)	0.378
	low (0.01-1.0)	587	1.35 (0.87, 2.11)	0.190
	zero (0)	113	3.38 (1.70, 6.84)	<0.001
**Mean Fluid Balance Change (ml/h)**	neutral (-20.83 - 20.83)	177	Reference	
	strong negative (<-62.5)	344	0.43 (0.26, 0.69)	<0.001
	light negative (-62.5 - -20.84)	222	0.52 (0.33, 0.82)	0.006
	positive (>20.83)	207	2.04 (1.26, 3.35)	0.004
**Age (years)**		950	1.03 (1.01, 1.04)	<0.001
**Sex**	Female	320	Reference	
	Male	630	0.81 (0.59, 1.13)	0.215
**BMI**		950	0.99 (0.96, 1.02)	0.386
**Emergency Admission**	False	361	Reference	
	True	589	1.98 (1.42, 2.80)	<0.001
**APACHE score at admission**		950	1.03 (1.00, 1.05)	0.035
**SOFA score at start of CRRT**		950	1.03 (0.99, 1.08)	0.151
**Invasive Ventilation at start of CRRT**		950	1.00 (0.66, 1.53)	1.000
**Noradrenaline increase above 0.1 mcg/kg/min**	False	288	Reference	
	True	662	1.16 (0.80, 1.67)	0.443
**Duration of CRRT session**		950	1.00 (1.00, 1.00)	<0.001

b

Variable		N	Odds ratio (95% CI)	p
**Early Increase of net Ultrafiltration Rate (ml/kg)**		802	1.00 (0.99, 1.01)	0.769
**Mean Fluid Balance Change (ml/h)**	neutral (-20.83 - 20.83)	164	Reference	
	strong negative (<-62.5)	338	0.35 (0.22, 0.54)	<0.001
	light negative (-62.5 - -20.84)	206	0.45 (0.28, 0.72)	<0.001
	positive (>20.83)	94	2.12 (1.23, 3.71)	0.007
**Age (years)**		802	1.03 (1.02, 1.04)	<0.001
**Sex**	Female	283	Reference	
	Male	519	0.95 (0.68, 1.34)	0.770
**BMI**		802	1.00 (0.97, 1.03)	0.834
**Emergency Admission**	False	312	Reference	
	True	490	1.93 (1.35, 2.80)	<0.001
**APACHE score at admission**		802	1.01 (0.99, 1.04)	0.253
**SOFA score at start of CRRT**		802	1.02 (0.97, 1.07)	0.478
**Invasive Ventilation at start of CRRT**		802	0.95 (0.60, 1.50)	0.810
**Duration of CRRT session**		802	1.00 (1.00, 1.00)	0.002

eFigure 5. Logistic Regression including Noradrenalin Increase (a) and for Early Increase in NUF Rate (b) and 28-Day Mortality.

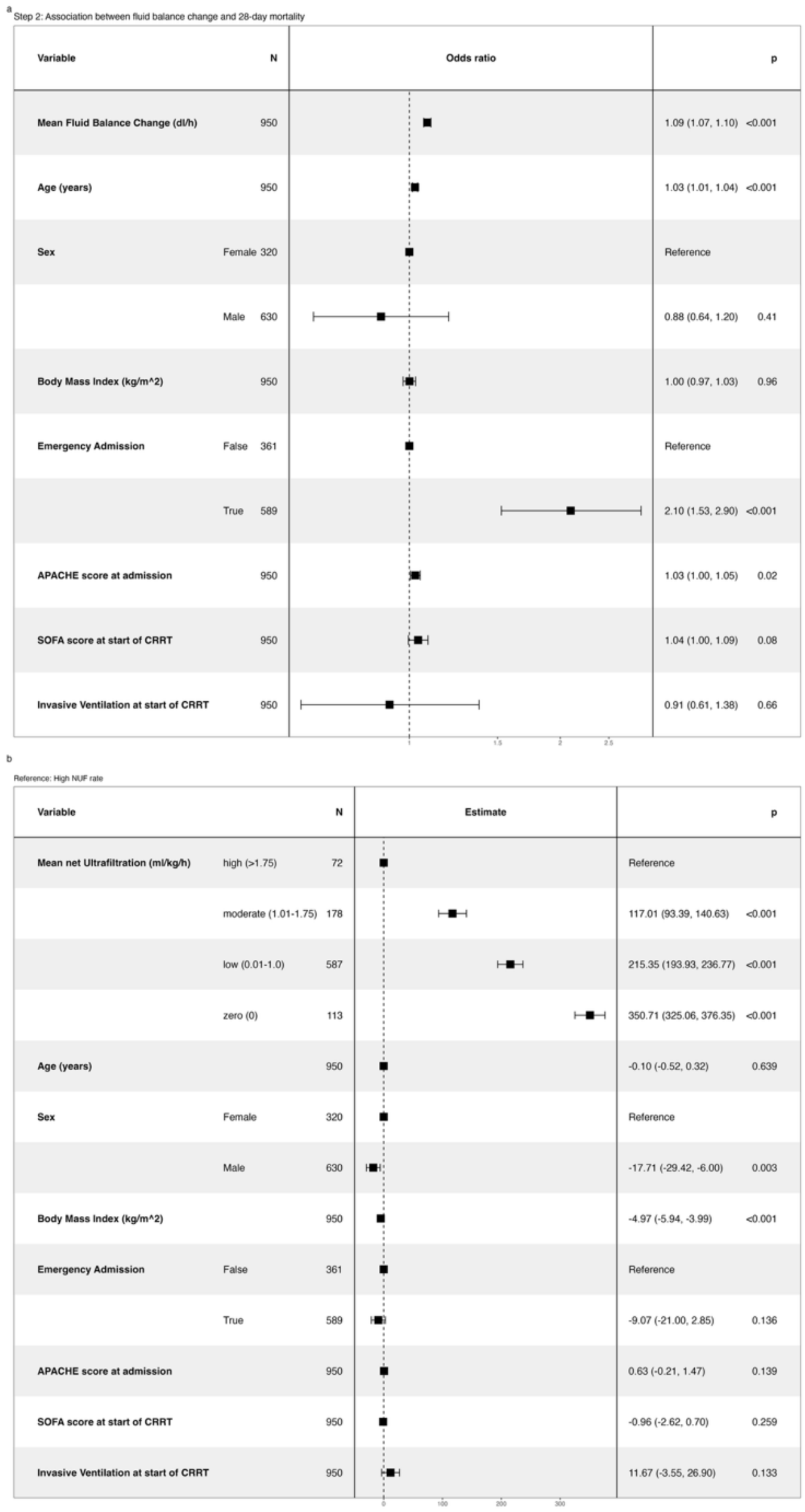

eFigure 6. Prerequisit Regression Models for Mediation Analysis.
(a) Association between NUF rate group and fluid balance change group.
(b) Association between fluid balance change group and 28-day mortality

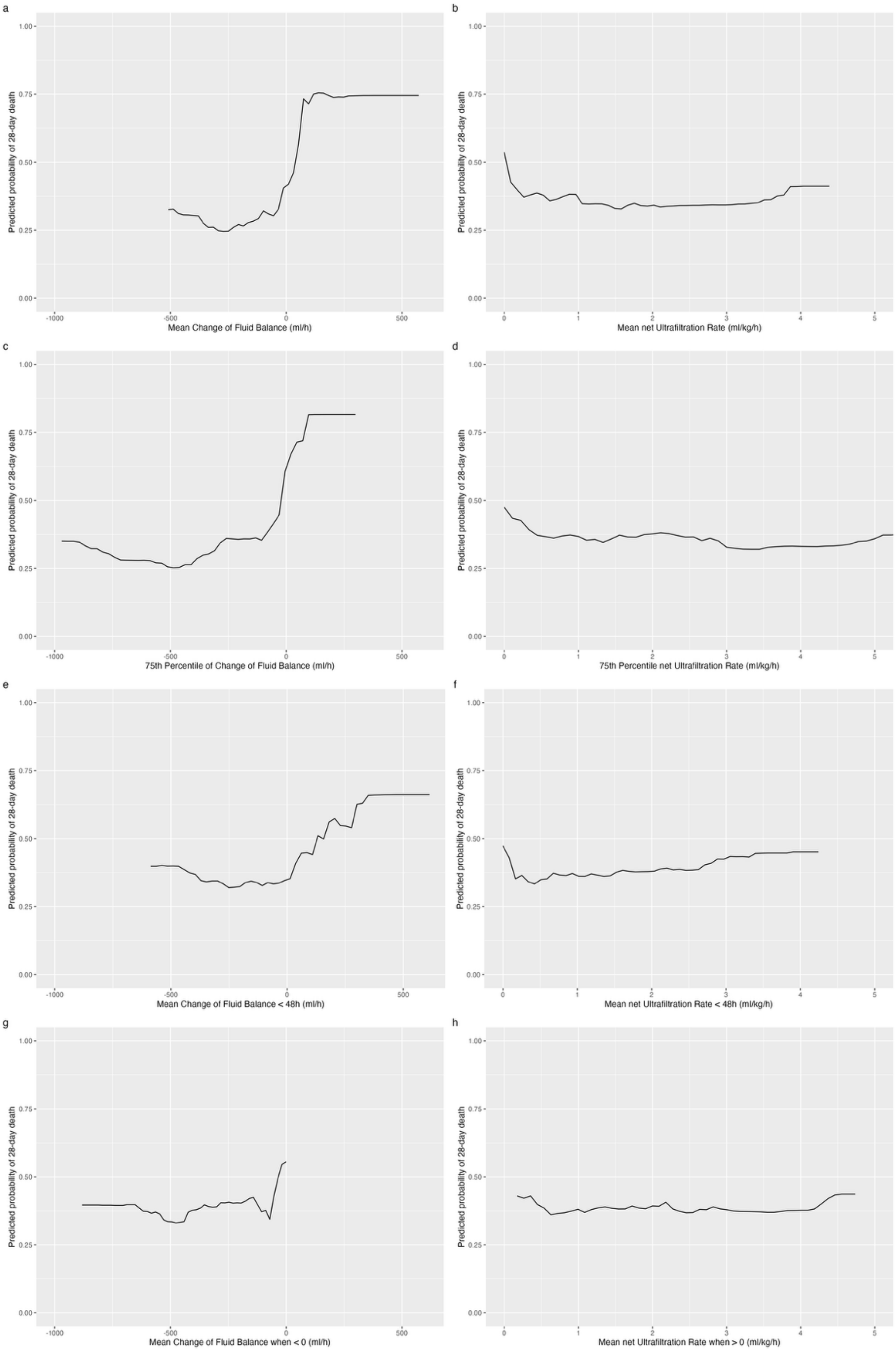

eFigure 7. Partial Dependence Plots (PDP) from Random Forest Models.

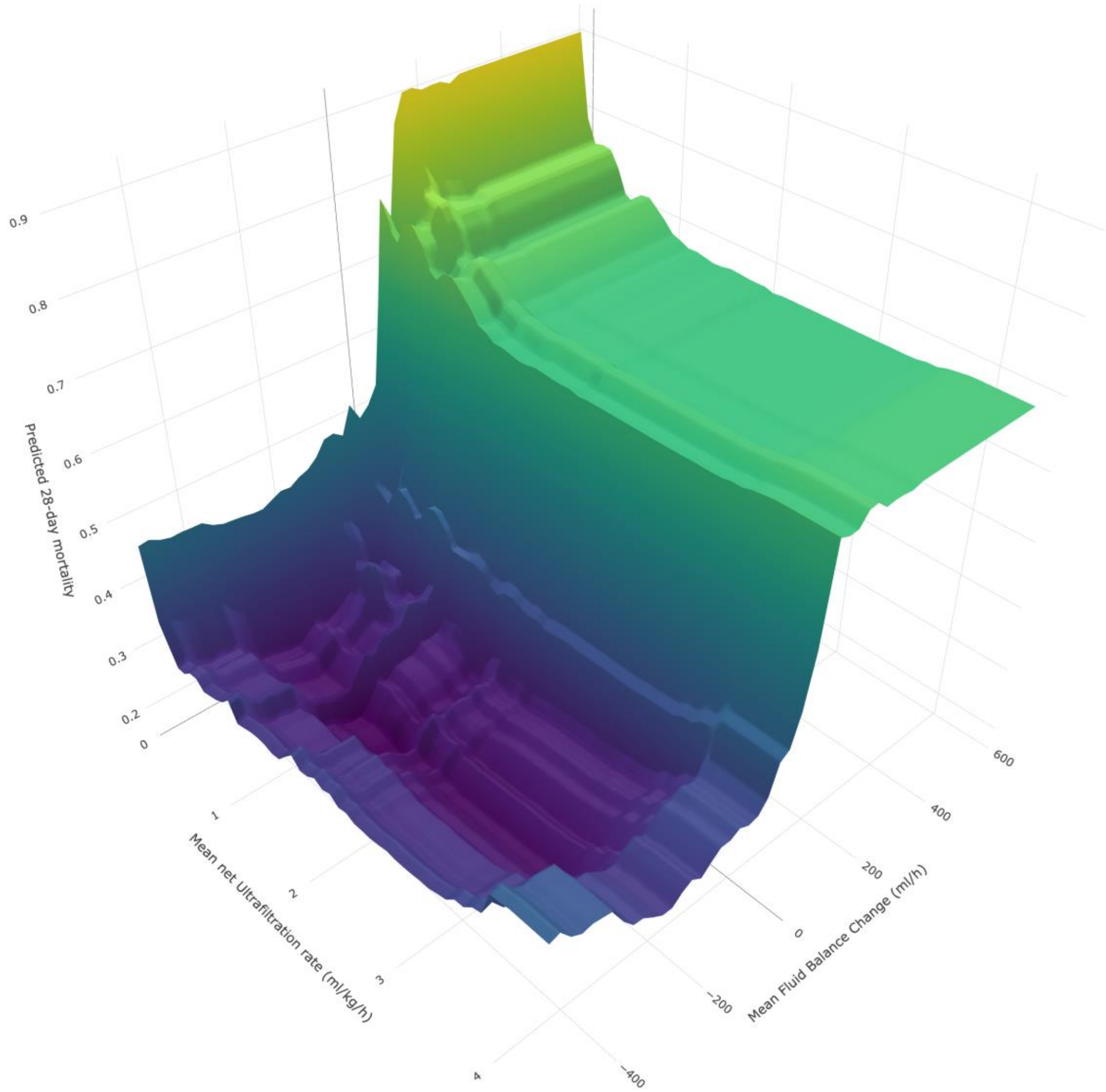

eFigure 8. Combined Effect of NUF Rate and Fluid Balance Change on Predicted 28-Day Mortality.

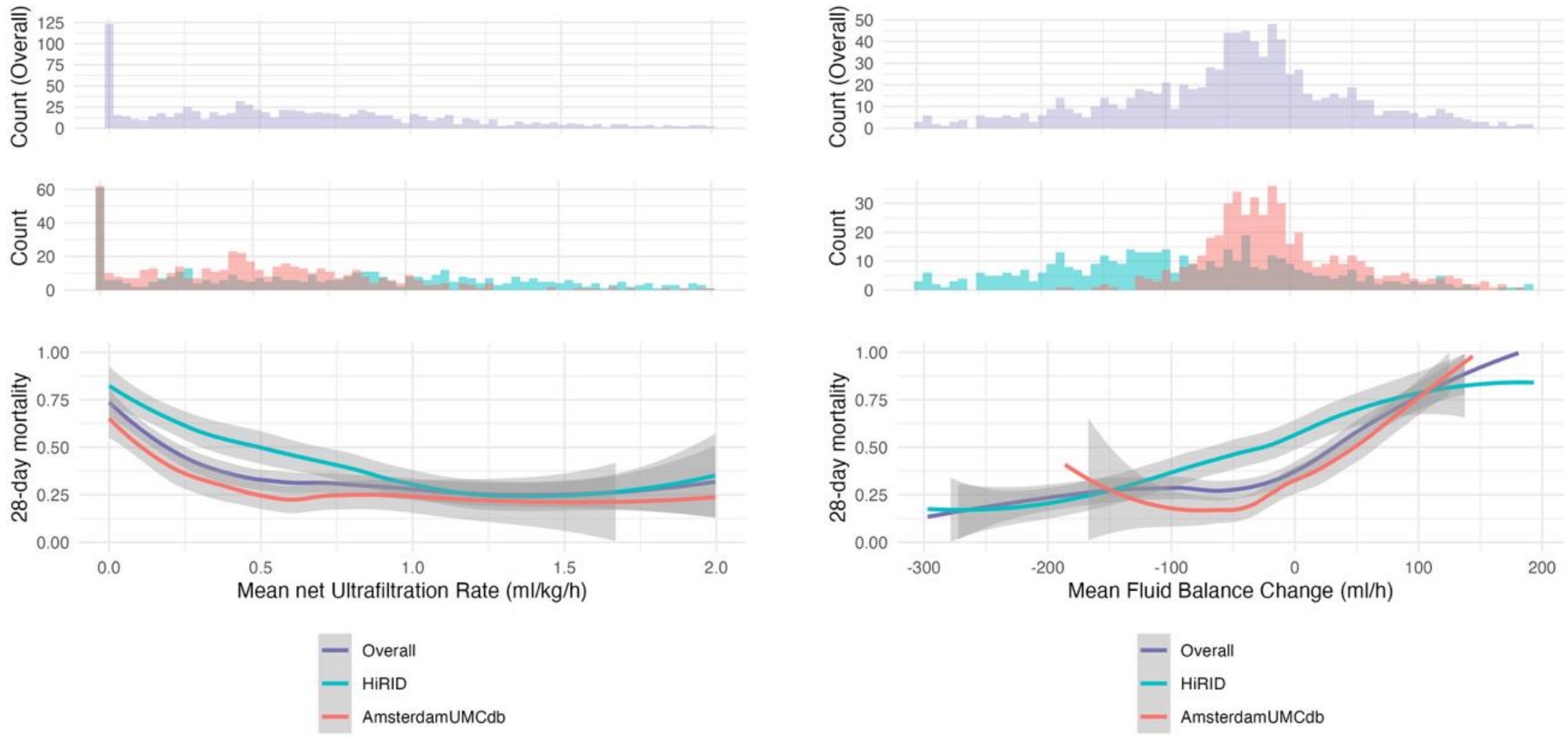

eFigure 9: Analysis by Database

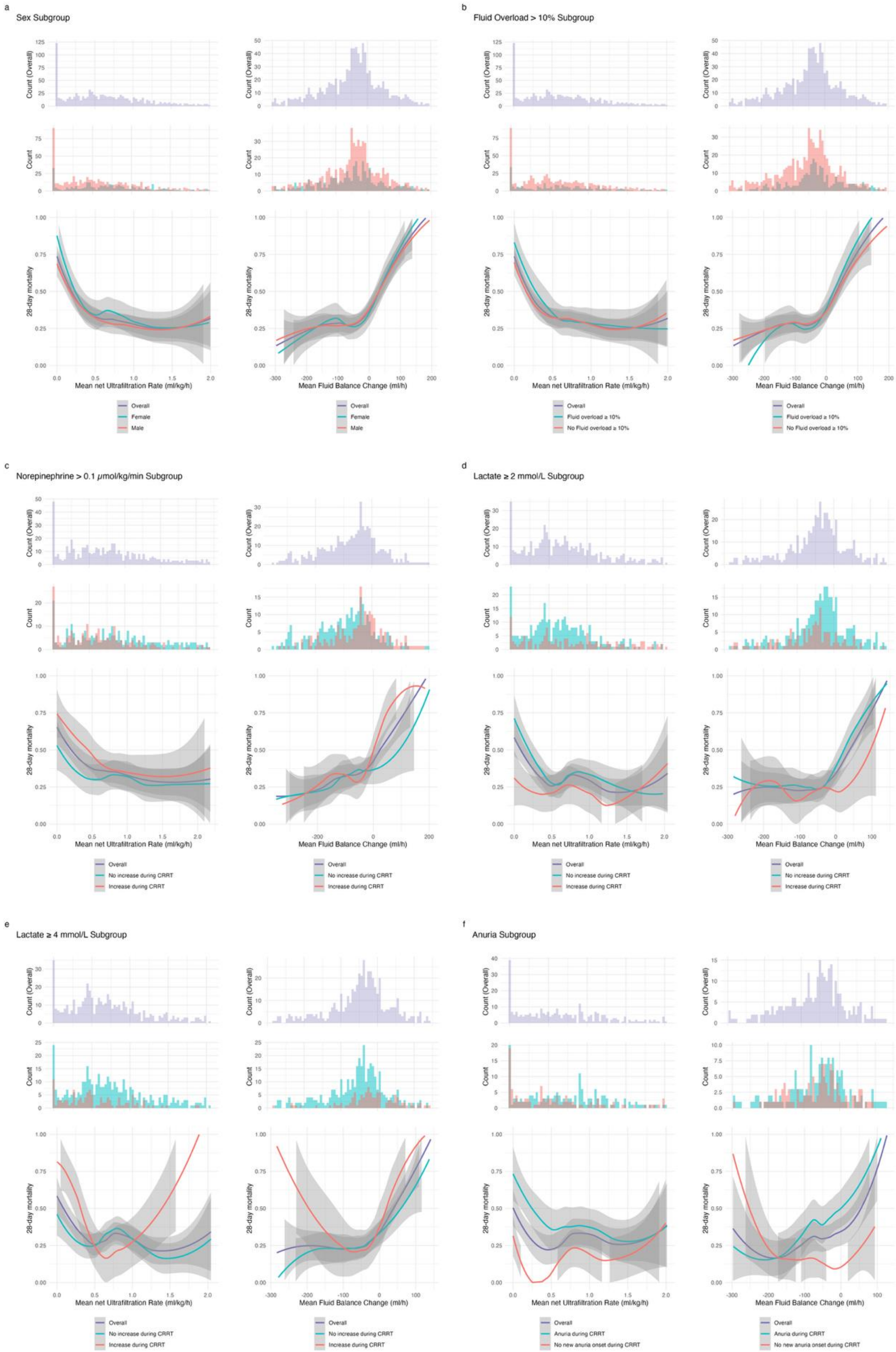

eFigure 10: Analyses by different subgroups

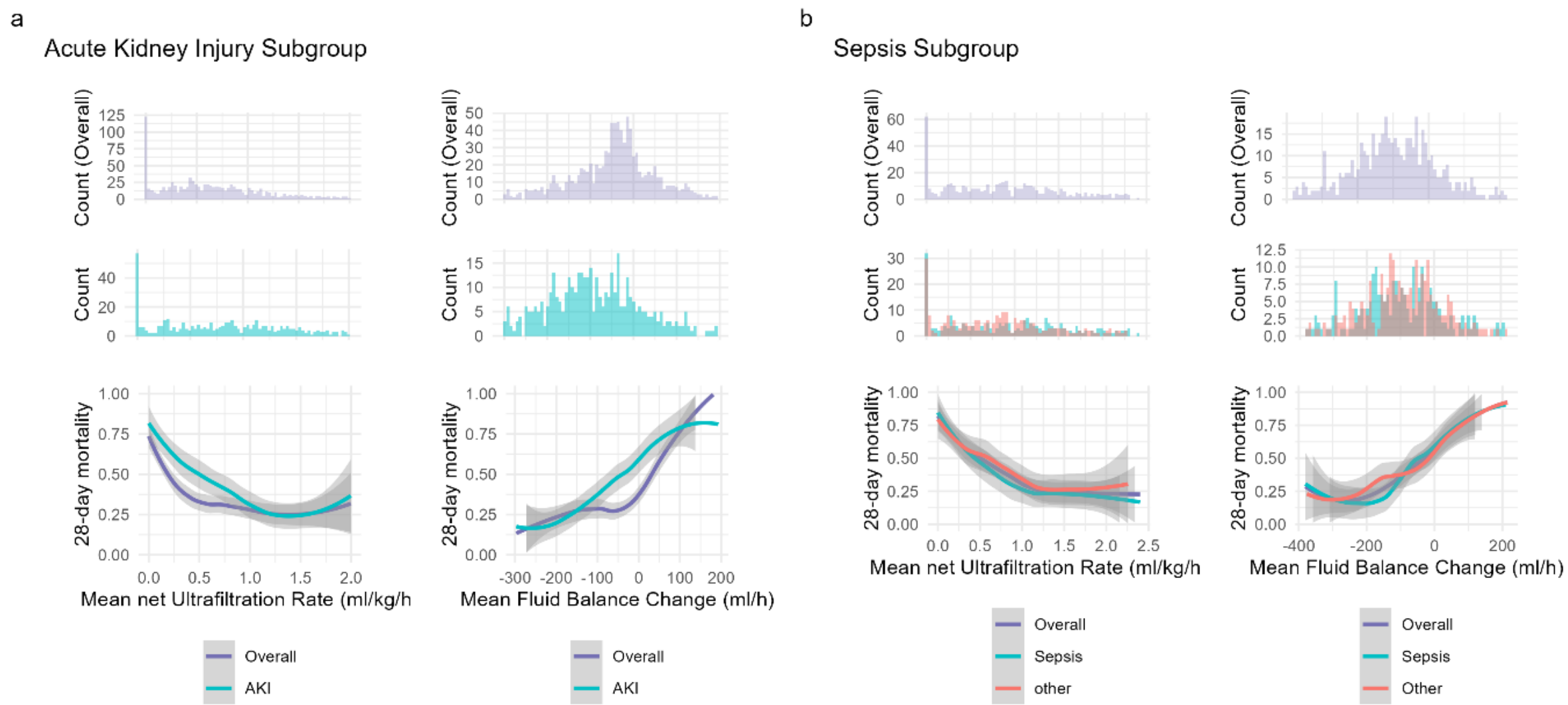

eFigure 11: Analyses by Diagnosis Subgroups

eTable 1: Population Characteristics in different Net Ultrafiltration Groups

Characteristic	**Overall** N = 973[1]	Ultrafiltration Group of Mean Net Ultrafiltration (ml/kg/h)			
		**high (>1.75)** N = 74[1]	**moderate (1.01-1.75)** N = 181[1]	**low (0.01-1.0)** N = 601[1]	**zero (0)** N = 117[1]
Female Sex	328 (33.7%)	38 (51.4%)	71 (39.2%)	189 (31.4%)	30 (25.6%)
Age (years)	64.0 (54.0-74.0)	64.0 (54.0-72.0)	64.0 (55.0-71.0)	64.0 (54.0-74.0)	64.0 (54.0-74.0)
Height (cm)	174 (165-180)	168 (161-175)	170 (164-179)	174 (165-183)	174 (169-184)
Weight (kg)	80 (70-94)	74 (63-84)	78 (68-90)	84 (74-94)	76 (74-85)
Body Mass Index (kg/m^2)	26.8 (23.9-30.7)	25.2 (23.8-29.3)	26.0 (23.7-31.0)	27.5 (24.4-30.7)	25.0 (23.8-27.8)
Emergency Admission	611 (62.8%)	61 (82.4%)	123 (68.0%)	347 (57.7%)	80 (68.4%)
Reason for ICU-Admission					
Cardiovascular or Vascular	242 (24.9%)	18 (24.3%)	49 (27.1%)	145 (24.1%)	30 (25.6%)
Cardiovascular or Vascular Surgery	219 (22.5%)	11 (14.9%)	47 (26.0%)	147 (24.5%)	14 (12.0%)
Other medical	211 (21.7%)	13 (17.6%)	32 (17.7%)	137 (22.8%)	29 (24.8%)
Respiratory	86 (8.8%)	17 (23.0%)	24 (13.3%)	36 (6.0%)	9 (7.7%)
Gastrointestinal	65 (6.7%)	5 (6.8%)	10 (5.5%)	39 (6.5%)	11 (9.4%)
Gastrointestinal Surgery	60 (6.2%)	6 (8.1%)	9 (5.0%)	35 (5.8%)	10 (8.5%)
Other surgical	59 (6.1%)	4 (5.4%)	6 (3.3%)	41 (6.8%)	8 (6.8%)
Unknown	31 (3.2%)	0 (0.0%)	4 (2.2%)	21 (3.5%)	6 (5.1%)
APACHE II Score at ICU-Admission	25 (20-31)	27 (22-33)	28 (22-32)	24 (20-30)	26 (21-32)
SOFA Score at start of CRRT	11 (9-14)	13 (9-15)	12 (10-15)	11 (9-13)	11 (9-14)
Invasive Ventilation at Start of CRRT	791 (81.3%)	62 (83.8%)	156 (86.2%)	477 (79.4%)	96 (82.1%)
Noradrenaline at Start of CRRT (mcg/kg/min)	0.1 (0.0-0.3)	0.0 (0.0-0.1)	0.0 (0.0-0.2)	0.1 (0.0-0.3)	0.2 (0.1-0.6)
Lactate at Start of CRRT (mmol/l)	2.6 (1.5-5.3)	2.1 (1.3-4.0)	2.2 (1.3-4.3)	2.5 (1.5-4.7)	6.6 (3.0-11.9)
Acute Kidney Injury	465 (93.9%)	68 (97.1%)	134 (96.4%)	210 (91.7%)	53 (93.0%)
End-Stage Kidney Disease	30 (6.1%)	2 (2.9%)	5 (3.6%)	19 (8.3%)	4 (7.0%)
Post-Surgical Acute Kidney Injury	141 (28.5%)	19 (27.1%)	54 (38.8%)	62 (27.1%)	6 (10.5%)
Sepsis	259 (52.3%)	32 (45.7%)	66 (47.5%)	133 (58.1%)	28 (49.1%)
Fluid Balance before CRRT (L)	6.9 (8.6)	6.4 (8.7)	6.8 (8.6)	7.0 (8.7)	6.9 (7.9)
Positive Fluid Balance before CRRT:	799 (84.3%)	58 (80.6%)	145 (81.5%)	501 (85.6%)	95 (84.1%)
Fluid Overload ≥ 5% (kg body weight)	489 (50.3%)	32 (43.2%)	97 (53.6%)	306 (50.9%)	54 (46.2%)
Fluid Overload ≥ 7% (kg body weight)	407 (41.8%)	30 (40.5%)	80 (44.2%)	251 (41.8%)	46 (39.3%)
Fluid Overload ≥ 10% (kg body weight)	273 (28.1%)	21 (28.4%)	52 (28.7%)	166 (27.6%)	34 (29.1%)
ICU Length of Stay (days)	10.5 (5.8-20.2)	8.3 (5.2-14.2)	10.7 (6.2-19.8)	11.8 (7.1-24.8)	3.5 (2.3-6.6)
ICU Death	368 (37.8%)	7 (9.5%)	43 (23.8%)	231 (38.4%)	87 (74.4%)
28-day Death	377 (38.7%)	17 (23.0%)	45 (24.9%)	223 (37.1%)	92 (78.6%)

[1]n (%); Median (Q1-Q3); Mean (SD)

eTable 2: Landmark Analyses at 48 and 72 Hours After CRRT Initiation

N	Events	Model	Variable	HR (95% CI)	p-value
48 hours					
705	298	NUF only	NUF: high (>1.75)	1.07 (0.60–1.91)	0.814
705	298	NUF only	NUF: low (0.01-1.0)	0.92 (0.62–1.35)	0.662
705	298	NUF only	NUF: zero (0)	1.20 (0.79–1.84)	0.392
705	298	NUF + FB	NUF: high (>1.75)	1.07 (0.60–1.91)	0.823
705	298	NUF + FB	NUF: low (0.01-1.0)	0.86 (0.56–1.31)	0.486
705	298	NUF + FB	NUF: zero (0)	1.05 (0.64–1.71)	0.852
705	298	NUF + FB	FB: strong negative (<-62.5)	1.11 (0.71–1.73)	0.658
705	298	NUF + FB	FB: light negative (-62.5 - -20.84)	1.02 (0.58–1.78)	0.949
705	298	NUF + FB	FB: positive (>20.83)	1.29 (0.87–1.91)	0.206
72 hours					
664	257	NUF only	NUF: high (>1.75)	1.34 (0.74–2.42)	0.335
664	257	NUF only	NUF: low (0.01-1.0)	1.27 (0.85–1.90)	0.240
664	257	NUF only	NUF: zero (0)	1.40 (0.84–2.32)	0.197
664	257	NUF + FB	NUF: high (>1.75)	1.27 (0.70–2.31)	0.440
664	257	NUF + FB	NUF: low (0.01-1.0)	1.18 (0.76–1.83)	0.472
664	257	NUF + FB	NUF: zero (0)	1.20 (0.68–2.11)	0.529
664	257	NUF + FB	FB: strong negative (<-62.5)	1.37 (0.86–2.19)	0.191
664	257	NUF + FB	FB: light negative (-62.5 - -20.84)	1.36 (0.81–2.27)	0.245
664	257	NUF + FB	FB: positive (>20.83)	1.57 (1.04–2.37)	0.032

Cox proportional hazards models. NUF and fluid balance exposure calculated as mean during the landmark window. Models adjusted for age, sex, BMI, emergency admission, and APACHE II score. The proportional hazards assumption was satisfied for all variables at both timepoints (global Schoenfeld test $p = 0.81$ at 48h; $p = 0.96$ at 72h). Reference categories: NUF moderate (1.01–1.75 ml/kg/h); fluid balance neutral (−20.83 to 20.83 ml/h).

eTable 3: Mediation Analysis

Comparison	ACME	ACME_CI	ACME_p	ADE	ADE_CI	ADE_p	Total_Effect	TE_CI	TE_p	Prop_Mediated	PM_CI	PM_p
High vs Moderate	0.9869	[0.9160, 1.0685]	0.748	0.9439	[0.8314, 1.0778]	0.382	0.9316	[0.8301, 1.0447]	0.208	0.1861	[-4.4889, 4.1516]	0.796
High vs Low	0.7685	[0.6928, 0.8451]	0.000	1.0647	[0.9289, 1.2464]	0.372	0.8182	[0.7380, 0.9089]	0.000	1.3123	[0.7137, 3.0401]	0.000
High vs Zero	0.8043	[0.6745, 0.9741]	0.024	0.6699	[0.5294, 0.8285]	0.000	0.5388	[0.4787, 0.6078]	0.000	0.3522	[0.0399, 0.6603]	0.024
Moderate vs Low	0.8812	[0.8407, 0.9186]	0.000	0.9844	[0.9112, 1.0659]	0.752	0.8675	[0.8095, 0.9352]	0.000	0.8898	[0.5129, 1.8526]	0.000
Moderate vs Zero	0.8116	[0.7270, 0.9054]	0.000	0.7075	[0.6050, 0.8215]	0.000	0.5742	[0.5230, 0.6305]	0.000	0.3763	[0.1742, 0.6061]	0.000
Low vs Zero	0.8123	[0.7688, 0.8588]	0.000	0.8308	[0.7480, 0.9285]	0.000	0.6749	[0.6217, 0.7321]	0.000	0.5287	[0.3634, 0.7676]	0.000

Mediation analysis showing the average causal mediation effect (ACME), average direct effect (ADE), and total effect (TE) of the exposure on the outcome. Proportion mediated refers to the percentage of the total effect explained by the mediation pathway. Error bars represent 95% confidence intervals (CI). Reported p-values test the null hypothesis of no effect for each estimate (ACME_p, ADE_p, TE_p, and PM_p).

eTable 4. Vasopressor and Lactate levels and trends, and occurrence of anuria

Characteristic	**Overall** N = 973[1]	Database **AmsterdamUMCDb** N = 478[1]	**HiRID** N = 495[1]	**p-value**[2]
Mean Noradrenaline dose (mcg/kg/min)	0.12 (0.20)	0.20 (0.26)	0.05 (0.06)	<0.001
Maximum Noradrenaline dose (mcg/kg/min)	0.21 (0.06-0.52)	0.47 (0.18-0.82)	0.12 (0.03-0.24)	<0.001
Mean Lactate level (mmol/l)	2.75 (2.26)	2.45 (1.77)	3.03 (2.62)	<0.001
Maximum Lactate level (mmol/l)	3.7 (2.3-7.4)	3.5 (2.3-6.0)	4.0 (2.3-8.7)	<0.001
Noradrenaline > 0.1 mcg/kg/min				<0.001
Elevated at start	451 (46%)	299 (63%)	152 (31%)	
Increased during CRRT	221 (23%)	88 (18%)	133 (27%)	
Never elevated	301 (31%)	91 (19%)	210 (42%)	
Lactate > 2 mmol/l				<0.001
Elevated at start	465 (48%)	167 (35%)	298 (60%)	
Increased during CRRT	342 (35%)	224 (47%)	118 (24%)	
Never elevated	166 (17%)	87 (18%)	79 (16%)	
Lactate > 4 mmol/l				<0.001
Elevated at start	256 (26%)	83 (17%)	173 (35%)	
Increased during CRRT	203 (21%)	124 (26%)	79 (16%)	
Never elevated	514 (53%)	271 (57%)	243 (49%)	
Onset of Anuria (< 100ml/24h)				0.003
Anuria at start	675 (69%)	336 (70%)	339 (68%)	
Anuria onset during CRRT	181 (19%)	72 (15%)	109 (22%)	
Never anuric	117 (12%)	70 (15%)	47 (9.5%)	

[1]Mean (SD); Median (Q1-Q3); n (%)

[2]Wilcoxon rank sum test; Pearson's Chi-squared test
